# Early Progression Prediction in Korean Crohn’s Disease Using a Korean-Specific PrediXcan Model

**DOI:** 10.3390/ijms26072910

**Published:** 2025-03-23

**Authors:** Tae-woo Kim, Soo Kyung Park, Jaeyoung Chun, Suji Kim, Chang Hwan Choi, Sang-Bum Kang, Ki Bae Bang, Tae Oh Kim, Geom Seog Seo, Jae Myung Cha, Yunho Jung, Hyun Gun Kim, Jong Pil Im, Kwang Sung Ahn, Chang Kyun Lee, Hyo Jong Kim, Sangsoo Kim, Dong Il Park

**Affiliations:** 1 Division of Gastroenterology, Department of Internal Medicine and Inflammatory Bowel Disease Center, Kangbuk Samsung Hospital, Sungkyunkwan University School of Medicine, Seoul 03181, Republic of Korea; saedktw5@naver.com (T.-w.K.); skparkmd@gmail.com (S.K.P.); 2 Medical Research Institute, Kangbuk Samsung Hospital, Sungkyunkwan University School of Medicine, Seoul 03181, Republic of Korea; 3 Department of Internal Medicine, Gangnam Severance Hospital, Yonsei University College of Medicine, Seoul 06273, Republic of Korea; j40479@gmail.com; 4 Department of Bioinformatics, Soongsil University, Seoul 06978, Republic of Korea; suz3548@gmail.com; 5 Department of Internal Medicine, College of Medicine, Chung-Ang University, Seoul 06973, Republic of Korea; gicch@cau.ac.kr; 6 Department of Internal Medicine, College of Medicine, Daejeon St. Mary’s Hospital, The Catholic University of Republic of Korea, Daejeon 34943, Republic of Korea; sangucsd@gmail.com; 7 Department of Internal Medicine, Dankook University College of Medicine, Cheonan 31116, Republic of Korea; kibaebang@gmail.com; 8 Department of Internal Medicine, Haeundae Paik Hospital, Inje University College of Medicine, Busan 48108, Republic of Korea; kto0440@paik.ac.kr; 9 Department of Internal Medicine, Digestive Disease Research Institute, Wonkwang University School of Medicine, Iksan 54538, Republic of Korea; medsgs@wonkwang.ac.kr; 10 Department of Internal Medicine, Kyung Hee University Hospital at Gang Dong, Kyung Hee University College of Medicine, Seoul 05278, Republic of Korea; clicknox@hanmail.net; 11 Division of Gastroenterology, Soonchunhyang University Cheonan Hospital, Cheonan 31151, Republic of Korea; yoonho7575@naver.com; 12 Department of Internal Medicine, Soonchunhyang University College of Medicine, Seoul 04401, Republic of Korea; medgun@schmc.ac.kr; 13 Department of Internal Medicine and Liver Research Institute, College of Medicine, Seoul National University, Seoul 03080, Republic of Korea; jpim0911@snu.ac.kr; 14 Functional Genome Institute, PDXen Biosystems Inc., Yongin 17111, Republic of Korea; kwangsung.ahn@gmail.com; 15 Department of Gastroenterology, Center for Crohn’s and Colitis, Kyung Hee University Hospital, Kyung Hee University College of Medicine, Seoul 02453, Republic of Korea; gidrlee@gmail.com (C.K.L.); hjkim@khmc.or.kr (H.J.K.)

**Keywords:** Crohn’s disease, machine learning, early progression, structuring, penetrating

## Abstract

Crohn’s disease (CD) is a chronic inflammatory disorder with potential progression to stricturing (B2) or penetrating (B3) phenotypes, leading to significant complications. Early identification of patients at risk for these complications is critical for personalized management. This study aimed to develop a predictive model using clinical data and a Korean-specific transcriptome-wide association study (TWAS) to forecast early progression in CD patients. A retrospective analysis of 430 Korean CD patients from 15 hospitals was conducted. Genotyping was performed using the Korea Biobank Array, and gene expression predictions were derived from a TWAS model based on terminal ileum data. Logistic regression models incorporating clinical and gene expression data predicted progression to B2 or B3 within 24 months of diagnosis. Among the cohort, 13.9% (60 patients) progressed to B2 and 16.9% (73 patients) to B3. The combined model achieved mean area under the curve (AUC) values of 0.788 for B2 and 0.785 for B3 progression. Key predictive genes for B2 included *CCDC154*, *FAM189A2*, and *TAS2R19*, while *PUS7*, *CCDC146*, and *MLXIP* were linked to B3 progression. This integrative model provides a robust approach for identifying high-risk CD patients, potentially enabling early, targeted interventions to reduce disease progression and associated complications.

## 1. Introduction

Crohn’s disease (CD) is a chronic inflammatory condition that affects all layers of the gastrointestinal tract and is characterized by a relapsing and remitting clinical course [1]. The behavior of CD can be classified into B1 (non-stricturing, non-penetrating), B2 (stricturing), and B3 (penetrating) according to the Montreal classification [2], and disease behavior is dynamic over time. Recent studies have reinforced this, demonstrating that patients with predominantly inflammatory disease (B1) at diagnosis are very likely to develop either stricturing (B2) or penetrating (B3) complications due to recurrent inflammation [3,4]. A study investigating the natural history of CD reported that 70% of patients eventually progressed to stricturing (B2) or penetrating (B3) disease behavior [5]. Identifying risk factors that predict early progression from B1 to B2/B3 is, therefore, critical for optimal disease management, and a treat-to-target strategy should be implemented to intervene during the B1 stage, before the disease progresses to B2 or B3.

Recently, we reported a study on CD that utilized PrediXcan, a tool that transforms genotype data into gene expression data [6]. PrediXcan is employed in transcriptome-wide association studies (TWAS) to assess the association between phenotypes and “imputed” gene expression, and it has been successfully applied in numerous cases [7]. However, as PrediXcan has primarily been developed and validated in European populations, its application to Korean and other Asian populations may yield different results, necessitating careful consideration of population-specific genetic backgrounds.

Herein, we developed a Korean PrediXcan model using data from Korean patients with CD and compared its performance with the original PrediXcan GTEx v7 model. Using the gene expression data identified through the Korean PrediXcan model, we constructed a predictive model for early progression to B2 or B3 disease behavior.

## 2. Results

Using the Korean PrediXcan model, we predicted gene expression values from the single nucleotide polymorphisms (SNPs) of each sample and evaluated their ability, alongside 10 clinical variables (CVs) (age, sex, misdiagnosed UC, appendectomy history, IBD family history, perianal involvement, anti-TNF therapy, smoking, extra-colonic involvement, and diagnosis location). The demographic and epidemiological characteristics of the 430 patients are presented in Table 1.

**Figure 1:**
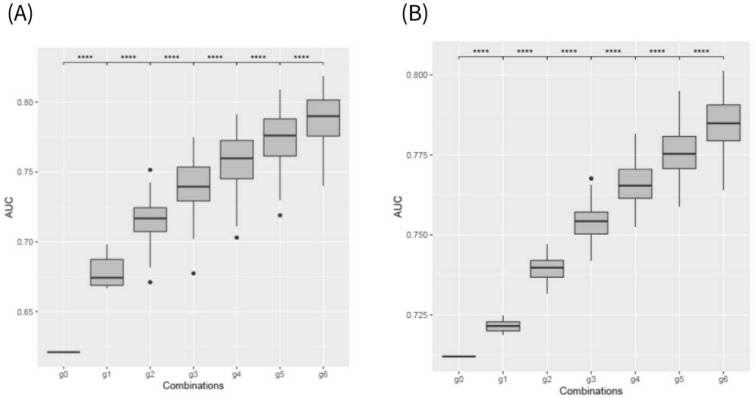
Improvement in AUC with the addition of each gene to CVs in each model: (**A**) B2 model (**B**) B3 model. g0 corresponds to “CVs only” (clinical variables only), while g1–g6 represent “CVs + 1 gene”, “CVs + 2 genes”, and so on. The boxplots illustrate the variation in AUC values obtained through leave-one-out cross-validation (LOOCV). Quadruple asterisks indicate statistical significance at *p* < 0.001 for pairwise *t*-tests between adjacent models.

We identified the top 10 genes with the highest predictive power by testing all those CVs in combination with individual gene expression values. When using only the CVs (the baseline models), the area under the curve (AUC) for the B2 model was 0.621, while the B3 model achieved an AUC of 0.712. These results indicate that the B3 model demonstrated relatively better predictive power using CVs alone (Figure 1).

We then progressively increased the number of genes in combinations, from two to six, and assessed performance. Our results demonstrated that adding more genes significantly increased the AUC in the gene combination groups (Figure 1). In the B2 model, the AUC improved from 0.678 with a single gene to 0.788 with six genes. Similarly, in the B3 model, the AUC increased from 0.722 to 0.785 as more genes were added. Supplementary Table S3 summarizes the AUC values with corresponding 95% confidence intervals for each combination of CVs and genes. When sensitivity and specificity were measured at the optimal cutoff values for each model, it was observed that both sensitivity and specificity decreased compared to using CVs alone. Furthermore, the Akaike information criterion (AIC) was measured to assess the model’s fit as the number of variables increased. It was observed that AIC decreased as more genes were included, indicating an improvement in model fit (Supplementary Table S3).

The selected genes from the best-performing models for each gene combination are listed in Supplementary Table S4. In addition, we conducted multivariate logistic regression on the final selected gene combinations, and the *p*-values reported in Table 2 represent the Wald test statistics, assessing the significance of each gene and CVs in the final models. In the B2 model, most gene variables had *p*-values below 0.01, indicating greater importance compared to CVs. Similarly, in the B3 model, most gene variables also had *p*-values below 0.01; however, CVs such as appendectomy history and anti-TNF therapy showed *p*-values below 0.001, indicating their significant roles in prediction. The model coefficients for these two CVs were positive and negative, respectively, suggesting that a history of appendectomy increased the likelihood of early progression, while the absence of anti-TNF therapy was associated with a higher risk of early progression.

## 3. Discussion

In this study, we utilized the PrediXcan model to infer tissue-specific gene expression from genotype data. Notably, we applied the Korean PrediXcan model, which was specifically developed using data from the Korean population, allowing for more accurate imputation of gene expression tailored to this population. By adjusting the number of predicted genes and incorporating clinical information, we successfully developed a highly predictive early progression model for CD, as reflected by mean AUC. In this model, six genes associated with disease behavior were identified: in the B2 progression group, *CCDC154*, *FAM189A2*, *TAS2R19*, *FCSK*, *SP1*, and *KCNIP1* were implicated, while in the B3 progression group, *PUS7*, *CCDC146*, *MLXIP*, *LRGUK*, *UROS*, and *TAFA1* were involved. Notably, clinical information played a more significant role in the B3 model compared to the B2 model.

Clinical indicators for predicting the progression of CD have been studied in many previous studies. Clinical factors associated with B2 or B3 included perianal disease, small bowel disease, smoking, prior steroid use, and anti-TNF therapy [8,9]. Additionally, ileocolonic disease and upper GI involvement were identified as factors related to B3 [10]. In our study, anti-TNF therapy and ileocolonic disease were also significant, consistent with findings from other studies [9,10]. However, prior studies have shown mixed results regarding the association between appendectomy and CD outcomes. For example, one study reported that prior appendectomy was positively associated with intestinal stricture risk (adjusted hazard ratio, 1.24; 95% CI, 1.13–1.36; *p* = 0.02) but inversely associated with perianal fistulation risk (adjusted hazard ratio, 0.75; 95% CI, 0.68–0.83; *p* = 0.002) [11]. However, other studies have found no significant differences in disease location, behavior, medication use, or need for intestinal resection between appendectomy and non-appendectomy groups [12]. In our study, the history of appendectomy demonstrated a significant correlation with B3 progression, highlighting the ongoing controversy and the need for further investigation into this association.

In previous studies, genes such as *CACNA1E*, *TNFSF15*, *NOD2*, *C5orf24*, *PCBD2*, *ATG16L1*, *PTGER4*, *C13orf31*, *CCR6*, and *CEBPB-PTPN1* were associated with B2 or B3 disease behavior [13,14,15,16]. In the current study, we identified new genes compared to those reported in previous research.

Our findings identified the association of the *SP1* (*specificity protein 1*) gene with B2 progression. *SP1* encodes a zinc finger transcription factor that binds to GC-rich motifs in numerous promoters [17]. This protein is involved in essential cellular processes, such as cell differentiation, proliferation, apoptosis, immune responses, DNA damage repair, and chromatin remodeling [18]. *SP1* has been previously implicated in IBD through its interaction with aryl hydrocarbon receptor (AhR), which provides negative feedback regulation of *AHRR*, a gene involved in methylation changes associated with IBD [19].

Through a Biogrid search, *SP1* was found to interact with *ATG16L1*, a gene previously linked to B2 and B3 disease phenotypes [13]. This interaction, identified through affinity capture-mass spectrometry, suggests their involvement in cellular processes such as autophagy. *ATG16L1* is crucial for autophagy and immune regulation, while *SP1* functions as a transcription factor that regulates multiple cellular processes [20]. The T300A variant of *ATG16L1* has been linked to increased susceptibility to CD, potentially affecting gene expression regulation involving *SP1*, particularly in inflammatory contexts [21].

Additionally, the *MLXIP* (*MLX interacting protein*) gene was identified as being related to B3 progression. *MLXIP* regulates the expression of glucose metabolism-related genes through its interaction with the transcription factor *MLX* [22]. The connection between CD and *MLXIP* may lie in the intersection between metabolic pathways and immune and inflammatory responses. Given *MLXIP*’s role in metabolic regulation, it is plausible that mutations in this gene could impair metabolic control or exacerbate inflammatory responses in CD. To date, no studies have directly explored the relationship between *MLXIP* and CD. However, future research on the interaction between metabolic and inflammatory pathways may elucidate the precise role of *MLXIP* and similar genes in the pathogenesis of CD.

One of the primary strengths of this study is the use of a Korean-specific PrediXcan model to predict the progression of CD. By tailoring the PrediXcan model specifically for the Korean population, the study addresses a significant gap in genetic research, which has historically focused on European populations. Additionally, the integration of gene expression data with CVs enhances the model’s predictive performance. The logistic regression model’s use of multiple genes, alongside clinical risk factors, demonstrates significant improvements in predictive accuracy, particularly with the inclusion of additional gene combinations. This approach highlights the potential for personalized medicine in CD management.

Despite the robust design, this study faces several limitations. The sample size, especially for the validation cohort, is relatively small, which may limit the generalizability of the findings to larger or more diverse populations. Although univariate analysis was conducted at the gene level to select the top 10 genes, we acknowledge that a rigorous machine learning-based feature selection approach would require larger datasets, preferably validated with external cohorts. Given our limited sample size, these results should be viewed as preliminary, emphasizing the need for further validation in larger, independent studies. Additionally, while the Korean PrediXcan model improves prediction accuracy for this specific population, its applicability to other Asian populations remains uncertain due to potential genetic heterogeneity. Moreover, the reliance on imputed gene expression data, rather than direct measurements, introduces potential biases. The complexity of the model, which combines numerous genetic and clinical factors, may also present challenges in practical clinical application, particularly in settings with limited resources or genetic testing infrastructure. Specifically, we note that caution is required when interpreting our findings and that future prospective studies or larger external cohorts would help validate our results and mitigate concerns regarding selection bias.

In conclusion, this study successfully developed an early progression model for CD using a Korean-specific PrediXcan model, identifying key genes associated with disease progression (B2 and B3) and demonstrating the added predictive value of CVs. These findings may help guide early therapeutic decisions, particularly in tailoring treatments to prevent complications in patients at higher risk. Further research is necessary to validate these findings in larger and more diverse populations.

## 4. Materials and Methods

### 4.1. Study Population

A total of 894 patients with inflammatory bowel disease (IBD) were enrolled from three Korean IBD cohorts: IMPACT, UC multiomics, and OACIS. The details of each cohort are described in previous studies. The IMPACT (identification of the mechanism of the occurrence and progression of CD through integrated analysis on both genetic and environmental factors) study [6,23,24,25], initiated in 2017, is a prospective multicenter study involving 16 university hospitals, collecting clinical data and biological specimens (blood, stool, and tissue) from newly diagnosed or monitored patients with CD. The UC multiomics study [6,24], started in 2020 at 14 university hospitals, gathered clinical data and biological samples (blood, stool, tissue, and saliva) from patients with ulcerative colitis (UC). The OACIS study [6,26], conducted from August 2016 to September 2019 across 18 university hospitals, is a prospective observational study that enrolled patients with moderate to severe CD or UC, aged over 18, who initiated CT-P13 therapy.

For the development of Korean PrediXcan model, we included 107 patients with CD, selected from the three cohorts of 894 patients with IBD, with both terminal ileum normal tissue RNA-seq data and genotype data. Patients were divided into two groups: the training group (n = 61) and the validation group (n = 46). The 107 CD samples originated from two RNA-seq datasets (61 and 46 samples), processed separately due to different library preparation methods. To avoid batch effects and ensure model robustness against technical variability, we used the 61-sample set for training and the 46-sample set for validation. For the early progression model, we included CD patients with genotype data, and the exclusion criteria were as follows: (1) cases with missing clinical data such as disease behavior; (2) follow-up period less than 2 years. After excluding these patients, 430 patients with CD out of the original 894 patients were included in the early progression model.

### 4.2. Genotyping

Details on sample genotyping are provided in a prior study [6]. Briefly, blood samples from the three cohorts were genotyped using the Korea Biobank Array. The Korean Reference genotype dataset was released by the National Biobank of Korea, Korea National Institute of Health, Osong, Korea (https://biobank.nih.go.kr/cadaver/cmm/main/engMainPage.do, accessed on 18 July 2019), and after quality control, 878 samples and 749,383 SNPs were retained for analysis. Imputation using the Korean reference panel and BEAGLE v5.0 (University of Washington, Weattle, WA, USA) resulted in 6,153,437 SNPs after further filtering for minor allele frequency and the Hardy–Weinberg equilibrium.

### 4.3. RNA-Seq

RNA-seq data from 107 patients with CD collected from the terminal ileum for the Korean PrediXcan model were processed using version 2.7.9a of STAR (Cold Spring Harbor Laboratory, Cold Spring Harbor, NY, USA) for mapping, and duplicate marking was performed with version 2.18.17 of Picard (Broad Institute, Cambridge, MA, USA). Afterward, quantification and normalization were conducted using version 2.4.2 of RNA-seQC (Broad Institute, Cambridge, MA, USA).

### 4.4. Development of the Korean PrediXcan Model

The detailed process for constructing the Korean PrediXcan model is outlined in the Supplementary Materials. The PrediXcan v7 model (University of Chicago, Chicago, IL, USA) has been developed primarily using data from European populations, specifically the DGN, GEUVADIS, and GTEx datasets. To create a model more suitable for the Korean population, we utilized the database of Korean CD patients to develop the Korean PrediXcan model (Supplementary Tables S1 and S2). For model development, we employed the 5-fold nested, cross-validation procedure as specified in the PrediXcan GTEx_v7 tutorial (https://github.com/hakyimlab/PredictDB_Pipeline_GTEx_v7 (accessed on 18 July 2019)), consistent with the original PrediXcan methodology. The training performance of the Korean model was assessed using the same metrics applied in the original PrediXcan v7 model. The Korean PrediXcan model developed in this study has been deposited in the publicly accessible repository Zenodo (http://zenodo.org/records/14992681 (accessed on 8 March 2025)).

### 4.5. Development of Early Progression Model Method

#### 4.5.1. Gene Expression

To develop an early progression model, we aimed to use gene expression data from the small intestine, specifically the terminal ileum. Gene expression in Korean patients with CD was predicted using the Korean PrediXcan model, which showed better performance than the original PrediXcan v7 model (Supplementary Materials). Both the B2 and B3 models predicted 1904 genes, and by selecting only protein-coding genes, a final set of 1380 genes were used as features for the early progression model.

#### 4.5.2. Prediction Model and Performance Evaluation

To develop the early progression model, we analyzed 430 patients with CD. Among these, patients who progressed from B1 to B2 within 24 months or who were already classified as B2 at diagnosis were categorized into the B2 group, resulting in a total of 60 patients in this group. Similarly, the B3 group included patients who progressed from B1 or B2 to B3 within 24 months or were initially diagnosed as B3, comprising a total of 73 patients. The remaining 297 patients, who stayed in the non-stricturing B1 group, were classified as the control group. We created two separate models: one for B2 and another for B3, combining the control set with each case group, resulting in 357 samples for the B2 model and 370 samples for the B3 model.

We used a logistic regression algorithm to develop the B2 and B3 early progression models. The features included 1380 genes predicted by the Korean PrediXcan model, as well as CVs such as age, sex, misdiagnosis as UC initially, appendectomy history, family history of IBD, perianal disease, anti-TNF therapy, smoking status, extraintestinal manifestations, and diagnostic location. Categorical variables among the clinical data were one-hot encoded using the get dummies function in Pandas. First, using the Korean PrediXcan model, which inherently accounts for linkage disequilibrium through elastic-net regression, we predicted expression levels for 1380 genes, then combined each gene individually with our CVs and measured the resulting AUC. The 10 genes that provided the highest AUC were designated as our “top 10 genes”. Next, we maintained the CVs and incrementally combined 2 to 6 of the top 10 genes to assess model performance. We performed exhaustive searches by evaluating all possible combinations of the top 10 genes: for instance, at CVs + 2 genes, 45 combinations (_10_C_2_) were assessed, and at CVs + 3 genes, 120 combinations (_10_C_3_) were considered. The *p*-values shown in Figure 1 were obtained using *t*-tests comparing the AUC values across these gene combination sets.

Model performance was evaluated using leave-one-out cross-validation to prevent overfitting with AUC as the evaluation metric. Additionally, the optimal cutoff values were determined for each model to calculate sensitivity and specificity. Furthermore, we monitored AIC to ensure that increasing model complexity remained justified and did not lead to excessive overfitting, thus balancing predictive performance and model parsimony.

## Figures and Tables

**Table 1:** Clinical characteristics of patients.

Characteristics		CD, n (%) (n = 430)
Age at diagnosis, year, median ± SD		26.9 ± 12.1
Gender, male		313 (72.8)
History of smoking		78 (18.1)
Family history of IBD		11 (2.6)
Disease location	Terminal ileum	118 (27.4)
	Colon	52 (12.1)
	Ileocolon	244 (56.7)
	Terminal ileum + upper GI	4 (0.9)
	Colon + upper GI	1 (0.2)
	Ileocolon + upper GI	11 (2.6)
Extra GI involvement	Arthritis	27 (6.3)
	Iritis/Uveitis	4 (0.9)
	Erythema nodosum/Stomatitis	4 (0.9)
Perianal disease		134 (31.2)
Appendectomy history		36 (8.4)
History of UC diagnosis		14 (3.3)
Anti-TNF-α treatment within 2 years		79 (18.4)
Disease behavior changes within 2 years	B1	297 (69.1)
	B2	60 (14.0)
	B3	73 (16.9)

CD, Crohn’s disease; GI, Gastrointestinal; IBD, Inflammatory bowel disease; SD, Standard deviation; UC, Ulcerative colitis.

**Table 2:** Influence of selected variables on predicting early progression to B2 or B3.

Model		Estimate	Standard Error	Z-Value	*p*-Value
B2	*CCDC154*	−5.446	1.710	−3.184	0.001
	*FAM189A2*	−0.842	0.315	−2.671	0.008
	*TAS2R19*	−1.012	0.358	−2.827	0.005
	*FCSK*	0.895	0.331	2.703	0.006
	*SP1*	2.109	0.669	3.151	0.002
	*KCNIP1*	−2.205	0.935	−2.359	0.018
B3	History of appendectomy	1.882	0.508	3.705	<0.001
	Anti-TNF use	−3.025	0.811	−3.730	<0.001
	Ileocolonic disease	1.412	0.608	2.323	0.020
	*PUS7*	−1.531	0.573	−2.671	0.008
	*CCDC146*	−0.910	0.301	−3.026	0.002
	*MLXIP*	−2.421	0.603	−4.013	<0.001
	*LRGUK*	0.752	0.317	2.373	0.018
	*UROS*	−1.476	0.533	−2.771	0.006
	*TAFA1*	−0.820	0.357	−2.298	0.022

## Data Availability

The original contributions presented in this study are included in the article/Supplementary Materials. Further inquiries can be directed to the corresponding authors.

## Supplementary Materials

The following supporting information can be downloaded at: https://www.mdpi.com/article/10.3390/ijms26072910/s1.

## References

[1] Ramos G.P., Papadakis K.A. (2019). Mechanisms of Disease: Inflammatory Bowel Diseases. Mayo Clin. Proc..

[2] Silverberg M.S., Satsangi J., Ahmad T., Arnott I.D., Bernstein C.N., Brant S.R., Caprilli R., Colombel J.F., Gasche C., Geboes K. (2005). Toward an integrated clinical, molecular and serological classification of inflammatory bowel disease: Report of a Working Party of the 2005 Montreal World Congress of Gastroenterology. Can. J. Gastroenterol. Hepatol..

[3] Roda G., Chien Ng S., Kotze P.G., Argollo M., Panaccione R., Spinelli A., Kaser A., Peyrin-Biroulet L., Danese S. (2020). Crohn’s disease. Nat. Rev. Dis. Primers.

[4] Louis E., Collard A., Oger A.F., Degroote E., El Yafi F.A.N., Belaiche J. (2001). Behaviour of Crohn’s disease according to the Vienna classification: Changing pattern over the course of the disease. Gut.

[5] Cosnes J., Cattan S., Blain A., Beaugerie L., Carbonnel F., Parc R., Gendre J.P. (2002). Long-term evolution of disease behavior of Crohn’s disease. Inflamm. Bowel Dis..

[6] Park S.K., Kim Y.B., Kim S., Lee C.W., Choi C.H., Kang S.B., Kim T.O., Bang K.B., Chun J., Cha J.M. (2022). Development of a Machine Learning Model to Predict Non-Durable Response to Anti-TNF Therapy in Crohn’s Disease Using Transcriptome Imputed from Genotypes. J. Pers. Med..

[7] Gamazon E.R., Wheeler H.E., Shah K.P., Mozaffari S.V., Aquino-Michaels K., Carroll R.J., Eyler A.E., Denny J.C., Nicolae D.L., GTEx Consortium (2015). A gene-based association method for mapping traits using reference transcriptome data. Nat. Genet..

[8] Tarrant K.M., Barclay M.L., Frampton C.M., Gearry R.B. (2008). Perianal disease predicts changes in Crohn’s disease phenotype-results of a population-based study of inflammatory bowel disease phenotype. Am. J. Gastroenterol..

[9] Lakatos P.L., Czegledi Z., Szamosi T., Banai J., David G., Zsigmond F., Pandur T., Erdelyi Z., Gemela O., Papp J. (2009). Perianal disease, small bowel disease, smoking, prior steroid or early azathioprine/biological therapy are predictors of disease behavior change in patients with Crohn’s disease. World J. Gastroenterol..

[10] Tang L.Y., Rawsthorne P., Bernstein C.N. (2006). Are perineal and luminal fistulas associated in Crohn’s disease? A population-based study. Clin. Gastroenterol. Hepatol..

[11] Cosnes J., Seksik P., Nion-Larmurier I., Beaugerie L., Gendre J.P. (2006). Prior appendectomy and the phenotype and course of Crohn’s disease. World J. Gastroenterol..

[12] Chen D., Ma J., Ben Q., Lu L., Wan X. (2019). Prior Appendectomy and the Onset and Course of Crohn’s Disease in Chinese Patients. Gastroenterol. Res. Pract..

[13] O’Donnell S., Borowski K., Espin-Garcia O., Milgrom R., Kabakchiev B., Stempak J., Panikkath D., Eksteen B., Xu W., Steinhart A.H. (2019). The Unsolved Link of Genetic Markers and Crohn’s Disease Progression: A North American Cohort Experience. Inflamm. Bowel Dis..

[14] Pernat Drobez C., Repnik K., Gorenjak M., Ferkolj I., Weersma R.K., Potocnik U. (2018). DNA polymorphisms predict time to progression from uncomplicated to complicated Crohn’s disease. Eur. J. Gastroenterol. Hepatol..

[15] Pernat Drobez C., Ferkolj I., Potocnik U., Repnik K. (2018). Crohn’s Disease Candidate Gene Alleles Predict Time to Progression from Inflammatory B1 to Stricturing B2, or Penetrating B3 Phenotype. Genet. Test. Mol. Biomarkers.

[16] Ditrich F., Blumel S., Biedermann L., Fournier N., Rossel J.B., Ellinghaus D., Franke A., Stange E.F., Rogler G., Scharl M. (2020). Genetic risk factors predict disease progression in Crohn’s disease patients of the Swiss inflammatory bowel disease cohort. Ther. Adv. Gastroenterol..

[17] Lee J.-A., Suh D.-C., Kang J.-E., Kim M.-H., Park H., Lee M.-N., Kim J.-M., Jeon B.-N., Roh H.-E., Yu M.-Y. (2005). Transcriptional activity of Sp1 is regulated by molecular interactions between the zinc finger DNA binding domain and the inhibitory domain with corepressors, and this interaction is modulated by MEK. J. Biol. Chem..

[18] Vellingiri B., Iyer M., Devi Subramaniam M., Jayaramayya K., Siama Z., Giridharan B., Narayanasamy A., Abdal Dayem A., Cho S.G. (2020). Understanding the Role of the Transcription Factor Sp1 in Ovarian Cancer: From Theory to Practice. Int. J. Mol. Sci..

[19] Hou J.J., Ma A.H., Qin Y.H. (2023). Activation of the aryl hydrocarbon receptor in inflammatory bowel disease: Insights from gut microbiota. Front. Cell Infect. Microbiol..

[20] Glas J., Konrad A., Schmechel S., Dambacher J., Seiderer J., Schroff F., Wetzke M., Roeske D., Torok H.P., Tonenchi L. (2008). The ATG16L1 gene variants rs2241879 and rs2241880 (T300A) are strongly associated with susceptibility to Crohn’s disease in the German population. Am. J. Gastroenterol..

[21] Gammoh N. (2020). The multifaceted functions of ATG16L1 in autophagy and related processes. J. Cell Sci..

[22] Hunt L.C., Xu B., Finkelstein D., Fan Y., Carroll P.A., Cheng P.F., Eisenman R.N., Demontis F. (2015). The glucose-sensing transcription factor MLX promotes myogenesis via myokine signaling. Genes. Dev..

[23] Park S.K., Kim H.N., Choi C.H., Im J.P., Cha J.M., Eun C.S., Kim T.O., Kang S.B., Bang K.B., Kim H.G. (2020). Differentially Abundant Bacterial Taxa Associated with Prognostic Variables of Crohn’s Disease: Results from the IMPACT Study. J. Clin. Med..

[24] Park S.K., Kim S., Lee G.Y., Kim S.Y., Kim W., Lee C.W., Park J.L., Choi C.H., Kang S.B., Kim T.O. (2021). Development of a Machine Learning Model to Distinguish between Ulcerative Colitis and Crohn’s Disease Using RNA Sequencing Data. Diagnostics.

[25] Kim H., Na J.E., Kim S., Kim T.O., Park S.K., Lee C.W., Kim K.O., Seo G.S., Kim M.S., Cha J.M. (2023). A Machine Learning-Based Diagnostic Model for Crohn’s Disease and Ulcerative Colitis Utilizing Fecal Microbiome Analysis. Microorganisms.

[26] Kim E.S., Kim S.K., Park D.I., Kim H.J., Lee Y.J., Koo J.S., Kim E.S., Yoon H., Lee J.H., Kim J.W. (2023). Comparison of the Pharmacokinetics of CT-P13 Between Crohn’s Disease and Ulcerative Colitis. J. Clin. Gastroenterol..

