# Supplementary material for: Early Progression Prediction in Korean Crohn’s Disease Using a Korean-Specific PrediXcan Model
Source: Int J Mol Sci. 2025 Mar 23;26(7):2910. doi: 10.3390/ijms26072910 (PMC11988338; doi:10.3390/ijms26072910)
Supplement: Supplementary file 1 [file ijms-26-02910-s001.zip › ijms-3453533-supplementary.pdf]

Supplementary Data

1. Development of Korean PrediXcan model

1.1 *Model training and comparing accuracy*

The original PrediXcan GTEx_v7 model was developed primarily using data from European populations, specifically the DGN, GEUVADIS, and GTEx datasets. For the development of the Korean PrediXcan model, we followed the methodology used in the original development of PrediXcan v7 model (https://github.com/hakyimlab/PredictDB_Pipeline_GTEx_v7). Gene expression was predicted using elastic-net regression, the same method used in the development of the original PrediXcan v7 model. To evaluate model performance and reduce the risk of overfitting, lambda parameters were tuned using nested cross-validation. Only local SNPs within 1 Mb of the target gene were used as features for prediction, effectively reducing the number of SNPs needed from an average of about 3,000 to a smaller, more relevant subset. Poorly predicted genes were filtered based on criteria consistent across models (nested cross-validation Pearson correlation > 0.1 and estimated p-value < 0.05).

The training performance of the Korean model was assessed using the same metrics applied in the original PrediXcan v7 model. The overall performance of predicted gene expression values was averaged across four test methods: (1) the average coefficient of determination (R^2) for the hold-out folds in nested cross-validation (test_R^2_avg); (2) the R^2 for the hold-out folds using the entire dataset for cross-validation; (3) the R^2 measured on the entire dataset with an elastic-net model trained on the entire dataset after selecting the lambda parameter; and (4) the Pearson correlation coefficient (PCC) for the hold-out folds in nested cross-validation. R^2 was calculated as 1 − sum((y_observed − y_predicted)**2) / sum((y_observed − mean(y_observed))**2). The test performance of the Korean model was also evaluated by examining the PCC between predicted and actual gene expression values in a validation group of 46 individuals.

1.2 *Model prediction accuracy*

1.2.1 Training accuracy

The small intestine terminal ileum (SI) model from PrediXcan v7 was used, and the training data performance was compared (Supplementary Table 1). The number of genes predicted by the European PrediXcan model was 3,113, while the Korean PrediXcan model predicted 1,908 genes. The average number of SNPs used for gene prediction was 32 for the European model and 54 for the Korean model. Performance metrics, including four indicators (test R^2, 10-fold cross-validation R^2, in-sample R^2, and rho), were used, and the average value of these metrics was compared for each gene. Except for the 10-fold cross-validation R^2 average, most training performances were higher in the Korean PrediXcan model compared to the European PrediXcan model.

Supplementary Table S1. European PrediXcan and Korean PrediXcan model training accuracy.

Mean	European PrediXcan	Korean PrediXcan
Sample	103	61
Gene	3,113	1,908
SNP	32	54
test_R2_avg	-35.651	0.061
cv_R2_avg	0.130	0.104
in_sample_R2	0.361	0.505

rho_avg	0.375	0.419

test_R^2_avg: the average coefficient of determination (R^2) in the test set, assessing how well the predicted gene expression explains the variance in observed gene expression. cv_R^2_avg: the average R^2 across cross-validation folds, measuring model performance consistency. in_sample_R^2: the R^2 value calculated within the training set, providing an estimate of model fit on the training data. rho_avg: average correlation between predicted and observed values on the hold-out folds during nested cross-validation. SNP, single nucleotide polymorphism.

1.2.2. Validation accuracy

To evaluate the performance of the constructed European and Korean PrediXcan models, gene expression values were predicted using the separate validation group (Supplementary Table 2), and their Pearson correlation coefficient (PCC) with the actual expression values was measured for comparison. First, all genes that could be predicted by both models were compared. The average PCC for the genes predicted by the Korean PrediXcan model was 0.05 higher than that of the European PrediXcan model. Additionally, the number of genes with a PCC greater than 0.5 was 56 higher in the Korean PrediXcan model than in the European model.

Supplementary Table S2. Average PCC of all genes predicted by the two models and the number of genes with PCC ≥ 0.5 in the validation group.

Genes & PCC		European PrediXcan	Korean PrediXcan
Number of genes		3,107	1,904
Mean of PCC		0.086	0.136
Range of PCC	0.8 ≦ PCC	2	9
	0.7 ≦ PCC < 0.8	8	23
	0.6 ≦ PCC < 0.7	38	46
	0.5 ≦ PCC < 0.6	46	72
Total		94	150

PCC, Pearson correlation coefficient, which measures the correlation between predicted and observed gene expression values, indicating the model's predictive accuracy.

Supplementary Table S3. Predictive power of combining CVs and genes in each model.

Model		Mean AUC (LOOCV)	95% CI	Alpha	Sensitivity	Specificity	AIC
B2	CVs only	0.621		0.113	0.983	0.296	306.16
	CVs+1 gene	0.678	0.670-0.686	0.140	0.900	0.492	296.15
	CVs+2 genes	0.715	0.710-0.720	0.257	0.633	0.768	288.59
	CVs+3 genes	0.740	0.737-0.743	0.148	0.867	0.609	280.47
	CVs+4 genes	0.759	0.756-0.761	0.218	0.716	0.747	274.79
	CVs+5 genes	0.774	0.772-0.777	0.122	0.883	0.616	267.38
	CVs+6 genes	0.788	0.785-0.790	0.102	0.9	0.619	261.41
B3	CVs only	0.712		0.182	0.781	0.559	332.32
	CVs+1 gene	0.722	0.720-0.723	0.195	0.712	0.657	330.15
	CVs+2 genes	0.740	0.739-0.741	0.195	0.753	0.684	324.73
	CVs+3 genes	0.754	0.753-0.755	0.204	0.699	0.721	312.39
	CVs+4 genes	0.766	0.765-0.767	0.265	0.698	0.781	307.64
	CVs+5 genes	0.776	0.775-0.777	0.223	0.739	0.723	301.42
	CVs+6 genes	0.785	0.784-0.786	0.165	0.821	0.690	297.84

CV, clinical variable; *AUC*, area under curve; *CI*, confidence interval; *LOOCV*, leave-one-out cross validation; *AIC*, Akaike information criterion.

Supplementary Table S4. Selected gene combinations from the best-performing model for each gene combination.

Model		Gene
B2	CVs+1 gene	*CCDC154*
	CVs+2 genes	*CCDC154, FAM189A2*
	CVs+3 genes	*CCDC154, FCSK, BRIX1*
	CVs+4 genes	*CCDC154, FAM189A2, FCSK, BRIX1*
	CVs+5 genes	*CCDC154, FAM189A2, TAS2R19, SP1, KCNIP1*
	CVs+6 genes	*CCDC154, FAM189A2, TAS2R19, SP1, KCNIP1, FCSK*
B3	CVs+1 gene	*DYNC2I1*
	CVs+2 genes	*DYNC2I1, LRGUK*
	CVs+3 genes	*PUS7, MLXIP, IL17Rc*
	CVs+4 genes	*PUS7, MLXIP, LRGUK, CCDC146*
	CVs+5 genes	*PUS7, MLXIP, LRGUK, CCDC146, UROS*
	CVs+6 genes	*PUS7, MLXIP, LRGUK, CCDC146, UROS, TAFA1*

CV, Clinical variable.
